# Virulence of Commercialized Fungal Entomopathogens Against Asian Longhorned Beetle (Coleoptera: Cerambycidae)

**DOI:** 10.1093/jisesa/ieaa006

**Published:** 2020-02-29

**Authors:** Eric H Clifton, Stefan T Jaronski, Ann E Hajek

**Affiliations:** 1 Department of Entomology, Cornell University, Ithaca, NY; 2 U.S. Department of Agriculture, Agricultural Research Service, Sidney, MT

**Keywords:** Asian longhorned beetle, entomopathogenic fungi, biological control, *Metarhizium*, *Beauveria*

## Abstract

Nine strains of five species of entomopathogenic hypocrealean fungi were tested against adults of the Asian longhorned beetle, *Anoplophora glabripennis* (Motschulsky). These strains have been developed as commercial biopesticide products in the United States, Brazil, South Korea, or the European Union (EU). *Metarhizium anisopliae* (Metschnikoff) (Hypocreales: Clavicipitaceae) ESALQ E-9 and *Metarhizium brunneum* (Petch) F52 (formerly *M. anisopliae* F52) (Hypocreales: Clavicipitaceae) killed 100% of treated beetles with the shortest survival times. Virulence differed among the five strains of *Beauveria bassiana* (Balsamo) (Hypocreales: Cordycipitaceae) tested, ranging from 0 to 77.3% mortality within 28 d. Two *Isaria fumosorosea* (Wize, 1904) (Hypocreales: Cordycipitaceae) (formerly *Paecilomyces fumosoroseus*) strains and the *Lecanicillium muscarium* (Petch) Zare & Gams (Hypocreales: Cordycipitaceae) strain used in Mycotal were not pathogenic to *A. glabripennis* adults. Within the entomopathogenic fungi tested, the *Metarhizium* strains may be the most appropriate for further evaluation.

More than 170 biopesticides based on entomopathogenic fungi (EPF) have been developed worldwide since the 1960s and, in 2007, approximately 75% of these were still in use or registered for use (Faria and Wraight 2007). Most of these use anamorphic hypocrealean species in the genera *Beauveria* and *Metarhizium*, but some biopesticides are based on *Isaria* and *Lecanicillium* (Chandler 2017). The market for all biopesticide products, including those with EPF, has had an annual growth rate of 15%, which is largely fueled by increased global spending on crop protection (Marrone 2014). Compared to chemical pesticides, mycoinsecticides are desirable because they pose minimal risk to beneficial nontargets like bees, parasitic wasps, and predatory beetles (Lacey et al. 2015). Developing EPFs as biopesticides that become authorized for use in a country can be a lengthy and expensive process. However, much research on the use of EPFs against a pest usually begins with testing strains that have not been developed as biopesticides (e.g., Dubois et al. 2008, Li et al. 2014). Although those types of studies are important initial steps, we suggest that EPF biopesticides that have been commercially developed should be included in bioassays as early as possible.

The Asian longhorned beetle, *Anoplophora glabripennis* (Motschulsky), is an invasive species in North America and Europe, and threatens urban and forest trees (Haack et al. 2010, Brabbs et al. 2015). The Asian longhorned beetle is a polyphagous pest known to feed on at least 10 genera of trees including *Acer*, *Populus*, *Salix*, *Ulmus*, and others (Hu et al. 2009). Eradication of Asian longhorned beetle from infested regions is difficult and expensive, requiring rigorous surveys of potential host trees, removal of infested trees and sometimes susceptible trees in the surrounding area, and, in some cases, injecting susceptible uninfested trees with a systemic insecticide (Hu et al. 2009). Additional management tools, including augmentative biological control, are desired for eradication programs. Previous studies have determined that EPF, including *Metarhizium* spp. and *Beauveria* spp., can infect and kill Asian longhorned beetle (Hajek et al. 2006, Dubois et al. 2008, Goble et al. 2014).

Our laboratory has previously tested 22 EPF strains against Asian longhorned beetle, of which three had been authorized for use in pest control in the United States (but one of these is no longer available) and one in Japan (Dubois et al. 2008, Goble et al. 2014). While Asian longhorned beetle was very susceptible to some of the tested EPFs, relatively few that have been tested are products that have been commercialized and registered for use in the United States. As described by many studies, there can be great variability in virulence among strains of the same EPF species (e.g., Dubois et al. 2008, Schapovaloff et al. 2014), thus comparing strains to find the most virulent is an important step. *Metarhizium brunneum* (petch) strain F52 (formerly *M. anisopliae* F52) (Hypocreales: Clavicipitaceae) has shown promise as a biological control agent for Asian longhorned beetle (Goble et al. 2015, 2016a,b, 2017; Gardescu et al. 2017; Clifton et al. 2019).

In the present study we challenged Asian longhorned beetle adults with nine strains of five species of EPF to compare their virulence. These EPFs have all been developed commercially and are registered for use in the United States or could potentially be registered in the United States because they are registered in another country.

## Materials and Methods

### Fungal Strains

We tested five fungal species in four genera, all of which have been commercially developed (Table 1). Some of the strains used in the current study are not registered in the United States, including *Beauveria bassiana* (Balsamo) (Hypocreales: Cordycipitaceae) strain ERL836, which has been commercialized for thrips management in South Korea, the *Lecanicillium muscarium* (Petch) Zare & Gams (Hypocreales: Cordycipitaceae) strain Ve-6, used as the active ingredient in Mycotal, and the ESALQ E-9 strain of *M. anisopliae* sensu lato, which is an active ingredient in Metarril, a biopesticide produced by Koppert Brazil. We tested *M. brunneum* strain F52 which was also referred to as *M. anisopliae* sensu lato until 2009 (Bischoff et al. 2009). Strains of *B. bassiana* (ANT-03 and ERL836), *Isaria fumosorosea* (Wize, 1904) (Hypocreales: Cordycipitaceae) (formerly *Paecilomyces fumosoroseus*) (Apopka Strain 97 and FE 9901), *M. brunneum* (F52), and *Metarhizium anisopliae* (ESALQ E-9) were unformulated, dry, conidial powders produced using solid-state fermentation by the USDA-ARS in Sidney, MT, following methods outlined in Jaronski and Jackson (2012). *Beauveria bassiana* (ATCC 74040) and *L. muscarium* (ARSEF 5128) were obtained from the USDA, ARSEF (Agricultural Research Service Collection of Entomopathogenic Fungal Cultures) in Ithaca, NY and were grown in solid culture for conidial production. Cultures of *B. bassiana* ATCC 74040 were grown on Sabouraud dextrose agar augmented with 1% yeast extract (SDAY) for 14 d before harvesting conidia. *Lecanicillium muscarium* was grown on selective media described in Kope et al. (2006) for 14 d before harvesting conidia. Dry conidial powder of *B. bassiana* strain GHA was provided by LAM International in Butte, MT.

**Table 1.** Entomopathogenic fungi tested against Asian longhorned beetles and information about commercialized products using the isolates tested as active ingredients

Fungus species (isolate)	Product (manufacturer)	Target pests^*a*^
*Beauveria bassiana* (ANT-03)	BioCeres (BioSafe Systems)	Aphids, whiteflies, thrips, plant bugs, beetles, weevils
*Beauveria bassiana* (GHA; ARSEF^*b*^ 6444)	BoteGHA & BotaniGard (Certis USA, Columbia, MD & BioWorks Inc., Victor, NY)	Whiteflies, thrips, aphids, psyllids, mealybugs, scarab beetles, plant bugs, weevils
*Beauveria bassiana* (ATCC^*c*^ 74040)	Naturalis (Troy Biosciences Inc., Phoenix, AZ)	Whiteflies, thrips, mites, aphids, lace bugs, weevils, wireworms, fruit flies, olive flies
*Beauveria bassiana* (ERL836)	Chongchaesak (LG-Chemical-affiliated FarmHannong, Korea)	Thrips
*Isaria fumosorosea* (Apopka Strain 97)	PFR-97 (Certis USA, Columbia, MD)	Whiteflies, aphids, thrips, mites, leaf miners, mealybugs, psyllids, plant bugs, weevils, rootworms, wireworms, caterpillars
*Isaria fumosorosea* (FE 9901)	NOFLY (Futureco Bioscience, Olèrdola, Barcelona, Spain)	Whiteflies, aphids, thrips, psyllids, mealybugs, leaf hoppers, plant bugs, weevils, grasshoppers, Mormon crickets, locusts, beetles, fungus gnats
*Lecanicillium muscarium* (Ve-6; ARSEF 5128)	Mycotal (Koppert Biological Systems, Berkel en Rodenrijs, Netherlands)	Whiteflies and thrips
*Metarhizium anisopliae* sensu lato (ESALQ E-9; ARSEF 925)	Metarril E9 (Koppert Brazil, São Paulo, Brazil)	Sugarcane root leafhopper
*Metarhizium brunneum* (F52; ARSEF 7711)	Met52 (Novozymes, Franklinton, NC)	Thrips, whiteflies, mites, weevils, ticks, chinch bugs

^*a*^According to online product descriptions from the manufacturers.

^*b*^ARSEF = Agricultural Research Service Collection of Entomopathogenic Fungal Cultures.

^*c*^ATCC = American Type Culture Collection.

One to two days before bioassays, the viability of strains was determined by spreading a dilute aqueous conidial suspension on Sabouraud dextrose agar. Germinated conidia were counted at 400× magnification 14–18 h after incubation at 25°C; all strains had >90% viability before use in bioassays.

### Experimental Insects

Adult Asian longhorned beetles were reared under USDA-APHIS permit in a quarantine facility adjacent to Cornell University, as described in the Supplementary Appendix of Goble et al. (2015). Prior to and during bioassays beetles were held in individual 473-ml clear plastic containers (PK16SC, Fabri-Kal, Kalamazoo, MI) in a 25°C:15°C day:night incubator with 14:10 (L:D) h. There was 100% relative humidity inside the sealed containers (i.e., visible condensation on the inner surface of the lid). Striped maple (*Acer pensylvanicum* L.) twigs with leaves removed were used for food. Beetles used for bioassays were 9–16 d after eclosion to adults (mean ± SE: 12.0 ± 0.1 d) when bioassays were begun.

### Bioassays Against *Anoplophora glabripennis*

We conducted immersion-exposure bioassays using methods developed by Dubois et al. (2008), with slight modifications. To compare fungal strains using a consistent exposure concentration, beetles were submerged for 5 s in suspensions of 1.0 × 10^8^ viable conidia per ml in 0.05% Tween. This concentration of conidia is a standard dose recommended by the International Centre of Insect Physiology and Ecology (ICIPE) for immersion-exposure bioassays with larger insects. A 1:1 sex ratio of females:males was used for each treatment. During each bioassay, beetles used for negative controls were exposed only to 0.05% Tween. This host is expensive and time-consuming to rear because it requires at least 9 mo to develop from egg to adult, and this situation limited sample sizes for a few EPF trials. The bioassays were repeated from 2 to 9 times on different dates for each strain. Totals of 22–44 insects were treated with each EPF, except the *Metarhizium* strains for which additional replicates were conducted. After the initial bioassays with *M. anisopliae* ESALQ E-9 resulted in short survival times for the exposed Asian longhorned beetle adults, we conducted more replicates with both *M. brunneum* F52 and *M. anisopliae* ESALQ E-9 for more detailed analysis of survival times.

For exposure, individual Asian longhorned beetle adults were placed in 50-ml tubes containing 11–13 ml of the conidial suspension and the tube was gently shaken for 5 s by hand. After immersion in the conidial suspensions, each adult was directly transferred to a container. Twigs used as food were replaced as necessary each week. Beetles were checked daily for mortality and experiments were ended 28 d after exposure. For two *B. bassiana* strains (ANT-03 and ERL836), few to no beetles had been killed by 28 d, but beetle behavior had significantly changed (noticeable lethargy and reduced feeding), so monitoring for these two EPF was extended to 42 d total. Dead beetles were kept in sealed containers and incubated at 20–25°C (0:24 [L:D] h) for up to 14 d to evaluate outgrowth by fungi.

### Statistical Analysis

Median survival times and SEs were calculated for adults receiving each treatment based on Kaplan–Meier survival distribution functions using the PROC LIFETEST statement in SAS 9.4 (SAS Institute Inc. 2019). For multiple comparisons of survival times among different EPF strains for which mortality was recorded, i.e., *Metarhizium* strains and *B. bassiana* GHA, the Cox proportional hazards model was performed in SAS 9.4 using the PROC PHREG statement. Contrasts between EPF strains were conducted using least-square means, adjusted with the Bonferroni correction. Controls were excluded from multiple comparisons because none of them died and, as a result, median survival times could not be calculated.

## Results

**Fig. 1.**
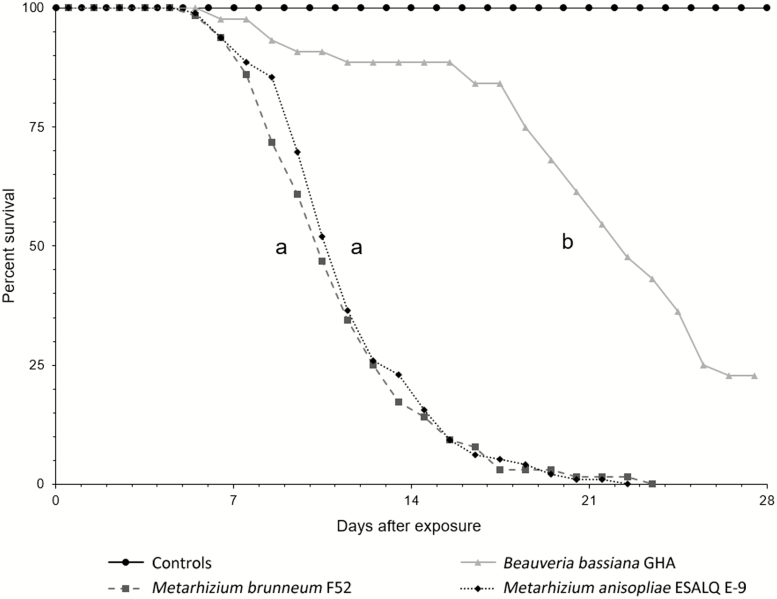
Survival graphs for Asian longhorned beetle adults exposed to *Metarhizium* isolates and *Beauveria bassiana* GHA (censored at 28 d). Median survival time was 11.3 ± 0.4 d for beetles exposed to F52, 11.7 ± 0.3 d for ESALQ E-9, and 21.4 ± 0.8 d for GHA. Letters denote significant differences between treatments based on the Cox proportional hazards model. Controls are included for comparison and had no mortality and therefore could not be included in the multiple comparisons.

*Metarhizium anisopliae* ESALQ E-9 and *M. brunneum* F52 each killed 100% of challenged beetles and 100% of the cadavers from these treatments had characteristic fungal outgrowth (Fig. 1; Table 2). *Beauveria bassiana* strain GHA killed 77.3% of beetles within 28 d and 100% of the cadavers had fungal outgrowth (Table 2; Fig. 1). *Beauveria bassiana* GHA (21.4 ± 0.8 d to death) killed beetles significantly slower than either ESALQ E-9 (11.7 ± 0.3 d) or F52 (11.3 ± 0.4 d) (Wald χ ^2^ tests; total α = 0.05 with Bonferroni correction; *P* < 0.0001) (Fig. 1). However, ESALQ E-9 and F52 killed beetles at similar speeds that were not significantly different (*P* = 0.6681) (Fig. 1).

**Table 2.** Percent mortality, median survival time in days (± SE), and % fungal outgrowth of laboratory-reared *A. glabripennis* inoculated by dipping adults in suspensions of 1.0 × 10^8^ viable conidia per ml

Fungus species	Isolate	No. bioassay replicates	No. insects	% killed by 28 d	Median survival time ± SE	Fungal outgrowth (%)
*Beauveria bassiana*	ANT-03	2	40	5.5%	–	100
*Beauveria bassiana*	ARSEF 6444 (GHA)	2	44	77.3%	21.4 ± 0.8b	100
*Beauveria bassiana*	ATCC 74040	2	40	0%	–	0.0
*Beauveria bassiana*	ERL836	2	28	0%	–	0.0
*Isaria fumosorosea*	Apopka Strain 97	2	40	0%	–	0.0
*Isaria fumosorosea*	FE 9901	2	40	0%	–	0.0
*Lecanicillium muscarium*	ARSEF 5128 (Ve-6)	2	22	0%	–	0.0
*Metarhizium anisopliae*	ARSEF 925 (ESALQ E-9)	6	96	100%	11.1 ± 0.3a	100
*Metarhizium brunneum*	ARSEF 7711 (F52)	9	92	100%	11.3 ± 0.4a	100
Control (0.05% Tween)	–	26^*a*^	104	0%	–	0.0

Bioassays were censored at 28 d. Letters next to the median survival times denote significant differences among treatments based on the Cox proportional hazards model. Fungal outgrowth refers to the % of cadavers with conidial outgrowth from the respective fungus used in the bioassay.

^*a*^Controls were always included in bioassays; hence, the large number of insects that were used.

Asian longhorned beetle adults exposed to *B. bassiana* strains ANT-03 or ERL836 responded noticeably slower and were feeding less 28 d after exposure, but only the ANT-03 strain killed any of the beetles (5.5%) within 28 d (Table 2). *Beauveria bassiana* strains ANT-03 and ERL836 caused 39.9% and 42.9% mortality, respectively, after 42 d. For those Asian longhorned beetle adults killed by *B. bassiana* ANT-03 and ERL836, 87.3% and 85.7% of the cadavers, respectively, had fungal outgrowth.

Both *I. fumosorosea* strains and *L. muscarium* Ve-6 did not kill any Asian longhorned beetle adults within 28 d after exposures and no control beetles died in the bioassays (Table 2).

## Discussion

*Metarhizium anisopliae* ESALQ E-9 and *M. brunneum* F52 killed 100% of exposed beetles and exhibited higher virulence than other EPF strains in the current study. Both EPF species are native to North America (Rehner and Kepler 2017). The results with *M. anisopliae* ESALQ E-9 are encouraging, but this strain, while commercial in Brazil, is not currently registered for use in the United States. *Metarhizium anisopliae* ESALQ E-9 has been studied for management of cattle ticks, spittlebugs, and the sugarcane borer, *Diatraea saccharalis* (Fabricius) (Lepidoptera: Crambidae), in Brazil, but has never before been tested against Asian longhorned beetle (Rossoni et al. 2014, Camargo et al. 2016). *Metarhizium brunneum* F52 already is registered for commercial sale in the United States.

Not all the EPF strains tested were pathogenic to Asian longhorned beetle adults. In general, the *B. bassiana* strains tested were less virulent to Asian longhorned beetle than *Metarhizium* strains. Bioassays by Dubois et al. (2008) compared six different strains of *B. bassiana*, including strain GHA. In agreement with the results from the present study, Dubois et al. (2008) reported that Asian longhorned beetle adults treated with *B. bassiana* strains had longer survival times than those exposed to different strains of *Metarhizium*. Furthermore, Asian strains of other *Beauveria* species (*Beauveria brongniartii* (Sacc.) Petch (ARSEF 6412), *Beauveria amorpha* (Höhn.) Minnis, S.A. Rehner & Humber (ARSEF 6827; formerly reported as *B. brongniartii*), and *Beauveria asiatica* S.A. Rehner & Humber (NBL 851; reported as *B. brongniartii*)) were more effective at killing Asian longhorned beetle than strains of *B. bassiana* (Dubois et al. 2008). At the time of the Dubois et al. (2008) study, *B. brongniartii* was not known as being endemic to North America (Rehner and Buckley 2005). No mycoinsecticides have been developed in North America using *B. brongniartii* as an active ingredient, although *B. asiatica* NBL 851 is used as the active ingredient in the Biolisa-Kamikiri (Idemitsu Kosan Co. Ltd., Tokyo, Japan) product sold in Japan for control of longhorned beetles (Faria and Wraight 2007, Rehner et al. 2011). After it was proven that *B. brongniartii* inhabits soils in North America (Rehner et al. 2011), Goble et al. (2014) conducted bioassays against Asian longhorned beetle adults, using methods similar to those in the current study, and found that *M. brunneum* F52 and *B. asiatica* (NBL 851) were more effective at killing Asian longhorned beetle adults than strains of *B. brongniartii* (ARSEF 6215 and ARSEF 10279) isolated from North American soils.

The two commercially developed strains of *I. fumosorosea* tested were not capable of infecting and killing Asian longhorned beetle adults. Commercially available mycoinsecticides containing strains of *I. fumosorosea* are typically used for sap-sucking pests (Chow et al. 2018), but we included this species in the present study because it has been reported infecting coleopteran pests like the citrus root weevil, *Pachnaeus litus* (Germar) (Coleoptera: Curculionidae) (Avery et al. 2016), yellowmargined leaf beetle, *Microtheca ochroloma* (Stål) (Coleoptera: Chrysomelidae) (Montemayor et al. 2016), and ambrosia beetles (Kushiyev et al. 2018). While screening EPF isolates for virulence against Asian longhorned beetle, Dubois et al. (2008) included a species of the same genus, *Isaria farinosa* (Holmsk.) Fr. (ARSEF 8411) (reported as *Paecilomyces farinosus* (Wize) A.H.S. Br. & G. Sm.), in the study because some Asian longhorned beetles in rearing had been killed by this EPF. However, no strains of this species are currently registered in any EPF products and, while it killed Asian longhorned beetle adults, the median survival time was 24.5 d (Dubois et al. 2008). Therefore, this strain of *I. farinosa* was not considered effective against Asian longhorned beetle when compared to strains of *Metarhizium* and *Beauveria*.

Mycoinsecticides that contain *Lecanicillium longisporum* (Petch) Zare & W. Gams (ARSEF 5126), *L. muscarium* (ARSEF 5128), and *L. lecanii* (formerly *Verticillium lecanii*) (Zimm.) Zare & W. Gams are available in the EU but not in North America. We tested *L. muscarium* against Asian longhorned beetle because using similar bioassay methods on the white pine weevil, *Pissodes strobi* (Peck) (Coleoptera: Curculionidae), with suspensions of 1.0 × 10^7^ viable conidia per ml, Kope et al. (2006) found that *L. longisporum* (Vertalec) and *L. muscarium* (Mycotal) caused median survival times of 9.1 ± 0.7 d and 7.9 ± 0.6 d, respectively. In addition, isolates of *L. muscarium* have been collected from great spruce bark beetles *Dendroctonus micans* (Kugelann) (Coleoptera: Curculionidae, Scolytinae) (Tanyeli et al. 2010). We found that *L. muscarium* did not kill any Asian longhorned beetle adults within 28 d and a limited assay with the strain of *L. longisporum* used in Vertalec (now discontinued by Koppert [its manufacturer]) produced the same results (E.H.C., unpublished data).

The labels and descriptions of EPF biopesticides include target pests (Table 1), but the potential host range is not always complete or well understood. For example, the EPF products using *M. brunneum* F52 and *B. bassiana* GHA mention beetles and weevils as target pests and both EPF strains killed Asian longhorned beetle adults. In contrast, the *B. bassiana* strains ATCC 74040 and ANT-03 also include beetles and weevils as target pests, but both of these EPF strains were less effective or failed to kill Asian longhorned beetle. *Metarhizium anisopliae* ESALQ E-9 is not promoted as killing beetles but this was one of the best strains tested against Asian longhorned beetle. The results in the present study demonstrate how some EPF are virulent to pests that are excluded from a biopesticide label, e.g., *M. anisopliae* ESALQ E-9 infecting Asian longhorned beetle.

Based on the results of the current study, EPFs in the genus *Metarhizium*, including *M. brunneum* F52 and *M. anisopliae* ESALQ E-9, could be recommended for further development as a tool for biological control of Asian longhorned beetle (Goble et al. 2016b, Gardescu et al. 2017). However, because F52 is already approved for commercial sale and is registered for use in the United States, eradication programs could consider this EPF among their tools. Certainly, field trials are needed with this fungus, or potentially other species or strains, to demonstrate practicality within an eradication program.
